# Health workforce incentives and dis-incentives during the COVID-19 pandemic: experiences from Democratic Republic of Congo, Nigeria, Senegal, and Uganda

**DOI:** 10.1186/s12913-024-10822-6

**Published:** 2024-04-03

**Authors:** Suzanne N. Kiwanuka, Ziyada Babirye, Steven N. Kabwama, Andrew K. Tusubira, Susan Kizito, Rawlance Ndejjo, Marc Bosonkie, Landry Egbende, Berthold Bondo, Mala Ali Mapatano, Ibrahima Seck, Oumar Bassoum, Mamadou MM Leye, Issakha Diallo, Olufunmilayo I. Fawole, Segun Bello, Mobolaji M Salawu, Eniola A Bamgboye, Magbagbeola David Dairo, Ayo Steven Adebowale, Rotimi . F Afolabi, Rhoda K. Wanyenze

**Affiliations:** 1 Department of Health Policy, Planning and Management, Makerere University College of Health Sciences School of Public Health, Kampala, P.O Box 7072, Uganda; 2 Kinshasa School of Public Health, Kinshasa, Democratic Republic of Congo; 3 Barumbu General Referral Hospital, Kinshasa, Democratic Republic of Congo; 4 The Cheikh-Anta-Diop University (UCAD), Dakar, Senegal; 5 Faculty of Public Health, College of Medicine, University of Ibadan, Ibadan, Nigeria

**Keywords:** Health workforce, Incentives, Dis-incentives, COVID-19

## Abstract

**Background:**

The COVID-19 pandemic presented a myriad of challenges for the health workforce around the world due to its escalating demand on service delivery. A motivated health workforce is critical to effectual emergency response and in some settings, incentivizing health workers motivates them and ensures continuity in the provision of health services. We describe health workforce experiences with incentives and dis-incentives during the COVID-19 response in the Democratic Republic of Congo (DRC), Senegal, Nigeria, and Uganda.

**Methods:**

This is a multi-country qualitative research study involving four African countries namely: DRC, Nigeria, Senegal, and Uganda which assessed the workplace incentives instituted in response to the COVID-19 pandemic. Key informant interviews (*n* = 60) were conducted with staff at ministries of health, policy makers and health workers. Interviews were virtual using the telephone or Zoom. They were audio recorded, transcribed verbatim, and analyzed thematically. Themes were identified and quotes were used to support findings.

**Results:**

Health worker incentives included (i) financial rewards in the form of allowances and salary increments. These motivated health workers, sustaining the health system and the health workers’ efforts during the COVID-19 response across the four countries. (ii) Non-financial incentives related to COVID-19 management such as provision of medicines/supplies, on the job trainings, medical care for health workers, social welfare including meals, transportation and housing, recognition, health insurance, psychosocial support, and supervision. Improvised determination and distribution of both financial and non-financial incentives were common across the countries. Dis-incentives included the lack of personal protective equipment, lack of transportation to health facilities during lockdown, long working hours, harassment by security forces and perceived unfairness in access to and inadequacy of financial incentives.

**Conclusion:**

Although important for worker motivation, financial and non-financial incentives generated some dis-incentives because of the perceived unfairness in their provision. Financial and non-financial incentives deployed during health emergencies should preferably be pre-determined, equitably and transparently provided because when arbitrarily applied, these same financial and non-financial incentives can potentially become dis-incentives. Moreover, financial incentives are useful only as far as they are administered together with non-financial incentives such as supportive and well-resourced work environments. The potential negative impacts of interventions such as service delivery re-organization and lockdown within already weakened systems need to be anticipated and due precautions exercised to reduce dis-incentives during emergencies.

**Supplementary Information:**

The online version contains supplementary material available at 10.1186/s12913-024-10822-6.

## Background

The health workforce constitutes an indispensable and costly input to any health system [1]. Indeed, the health workforce is fundamental to achieving health outcomes in the immediate term and ultimately universal health coverage and global health security [2]. The COVID-19 pandemic highlighted the critical importance of the health workforce in handling emergencies across the globe. By the end of 2022, over 6.4 million deaths were due to COVID-19 globally, with 222,276 deaths in Africa [3]. The unprecedented nature of the COVID-19 pandemic has posed manifold challenges for healthcare workers around the world [4]. These have manifested in terms of work overload [5], mental stress [6–8], infections [9, 10] and death [11]. The World Health Organization (WHO) estimates that between January 2020 to May 2021, approximately 80,000 to 180,000 health and care workers respectively may have died from COVID-19 globally (WHO 2021). These challenges and consequences have also resulted in health workers either absconding from duty [12] or in extreme circumstances resigning from the health profession [13] and opting for alternative professions [14]. During the 2014 Ebola epidemic, health workers in Liberia and Sierra Leone resorted to industrial actions due to poor pay, unsafe work conditions and death of their colleagues [15, 16] further compromising efforts to respond to the epidemic [17]. The training and skilling of health workers is an enormous investment of resources (both financial and time) for many countries. Hence, countries particularly those in resource-constrained settings cannot afford to lose their health workforce [18].

Health worker incentives refer to financial or non-financial mechanisms geared towards achieving a specific preferably positive behavioral change among health workers [19]. Non-financial incentives are those which do not involve financial worth or equivalent, to an individual [19]. These may include but are not limited to, promotion, continuing education, hospital infrastructure (working environment), availability of equipment and supplies, management and supervision, recognition or appreciation, job security and safety [20, 21]. Financial incentives on the other hand, may include allowances for overtime, risk allowances, insurance, and salary increments among others [20]. Available evidence revealed the need for a diversity of incentives to health workers [22]. Incentives for health workers have potential for motivating health workers [23], however, the nature of incentives during emergencies could differ from those offered routinely. During the Ebola outbreak response in Sierra Leone, Guinea, Liberia, and DRC, a broad package of incentives was instituted [24]. For example, in Sierra Leone, the incentives included provision of equipment, capacity building of health workers, social media support system, including trainings on how to deal with the stigma associated with being a health worker and a risk allowance [25]. In Serbia, Culafic (2020) highlighted the significance of health worker incentives during the COVID-19 response [26]. During COVID-19, several strategies were employed to motivate health workers. For instance, in Europe, compensation to families was offered following death of health workers e [27]. In sub Saharan Africa, where most countries have historically reported health workforce shortages [28], maldistribution [29, 30] and absenteeism [31], it is not known what strategies countries used to motivate and retain their health workforce during the COVID-19 pandemic. Moreover, most countries grappled with overwhelmed health systems characterized by overstretched facilities, insufficient drugs and stock-outs of personal protective equipment (PPE), which further exacerbated working conditions.

Poor implementation of incentives can result in dis-incentives for health workers. For instance, the perceived inadequacy, inconsistency, or unfair distribution of financial incentives can be a financial dis-incentive while inadequate staff, lack of supplies, unsafe environment and longer working hours may be a non-financial disincentive [32]. The inadequacy and perceived unfair distribution of incentives can generate dissatisfaction and overall loss of morale among health workers [33]. All these issues and their consequences are amplified during health emergencies and need to be interrogated further. In extreme circumstances, lack of incentives can result in health workforce migration [34]. Different approaches have been implemented to incentivize the health workforce across African countries during the COVID-19 pandemic. However, the scope of incentive strategies, their distribution, and the dis-incentives across the health workforce during the COVID-19 pandemic have not been well documented. Evidence on these incentive strategies and mitigating dis-incentives is critical for informing future outbreak response appropriate for already weakened health systems contexts. This study explored the scope of incentive strategies, experiences in their distribution and accompanying dis-incentives across the health workforce in DRC, Senegal, Nigeria, and Uganda during the COVID-19 response.

## Methods

### Study area

This study is part of a broader multi-country project that was conducted across two anglophone (Uganda and Nigeria) and two francophone (DRC, and Senegal) African countries, to assess and curate country experiences and their health system response to COVID-19.

### Democratic Republic of Congo (DRC)

The DRC is in Central Africa and occupies an area of 2,267,050 Km2 (875,313 sq. miles). It is the largest country in Sub-Saharan Africa [35]. The population of DR Congo is 95,675,956 as of October 02, 2022 [36]. DRC has 1.05 doctors, nurses and midwives per 1000 population. This is below the sub-Saharan African average of 1.2 per 1000 population, and far below the Sustainable Development Goals threshold of 4.45 doctors, nurses and midwives per 1000 population [37]. By December 31 2021, the DRC had reported 79,632 confirmed COVID-19 cases and 1,225 deaths, 35 of whom were health workers (Table 1). Only 0.25% of the population had been vaccinated [38].

**Table 1:** Description of Critical Health workforce indicators and COVID-19 outcomes across the four countries

Country (Population in millions)	DRC	Nigeria	Senegal	Uganda
**Doctors, nurses/midwives**	1.05 per 1000	2.0 per 1000	0.38 per 1000	2.58 per 1000
**Number of COVID-19 cases confirmed by end of December 2021**	79, 632	243,450	75,055	146,030
**COVID-19 deaths by end of December 2021**	1,225	3031	1890	3306
**Number of Health worker deaths due to COVID-19 by end of December 2021**	35	7*	5	37

* *Data on health worker deaths only available from one state out of 36*

*National data on health worker deaths couldn’t be accessed*

### Nigeria

Nigeria, in West Africa, has a total area of 910,770 Km2 (351,650 sq. miles) and its current population as of 2022 was 217,611,667 [39]. The Nigerian Health System is decentralized into three tier structures with responsibilities at federal, state and local government levels [40]. The country has 2.0 nurses, midwives, and doctors for every 1,000 people, less than the minimum recommended by the World Health Organization to provide adequate access to care [41]. By December 2021, Nigeria had reported 243,450 COVID-19 cases and 3031 deaths, 7 of whom were health workers from Rivers state, one of the 36 states in Nigeria [42] (Table 1).

### Senegal

Senegal is located on the bulge of West Africa [43]. It covers an area of 192,530 Km2 (74,336 sq. miles), with a population of 17,734,708 (2022) [44]. Senegal’s health care system operates with a three-tiered structure [45]. The number of doctors and nurses is 0.38 per 1,000 population in Senegal which is way below the 2.3 doctors and nurses per 1000 population that is recommended by the World Health Organization guidelines [46]. The cumulative confirmed cases by 31 December 2021 were 75,055 [47], and 1890 deaths, 5 of whom were health workers (Table 1).

### Uganda

Uganda is in Africa’s eastern region. It has 93,065 square miles, 76,100 of which are made up of land and 16,964 square miles as water sources [48]. As of October 2022 the population of Uganda was 48,985,049 [49]. The health system in Uganda comprises the national, regional, and local levels [50]. The number of doctors, nurses and midwives in Uganda was 2.58 per 1,000 population in the financial 2021/2022 [51]. By December 2021, Uganda had reported 146,030 COVID-19 cases and 3306 deaths, 37 of whom were health workers (Table 1).

### Study design

This was a qualitative descriptive cross sectional study which supported obtaining information about health worker incentive strategies and dis-incentives during the COVID-19 response across the four countries.

### Data collection

An unstructured key informant questionnaire guide (additional file 1) was developed with questions and probes interrogating health worker incentives and these were peer-reviewed across the research team and later translated into French for the franco-phone countries. The tool was pre-tested in Uganda and revised accordingly. Virtual interviews through the phone and Zoom were conducted with purposively selected key informants from November 2020 to March 2021 across the countries. Data were collected by 21 trained research assistants using the standardized pretested tools across the countries. Key informant interviews (*n* = 60) were conducted with managers in ministries of health (*n* = 17), policy makers (*n* = 18) and health workers (*n* = 25) to describe the health worker incentive strategies and dis-incentives during the COVID-19 response. Twelve (12) key informants were interviewed in DRC, 7 in Nigeria, 20 in Senegal, and 21 in Uganda. On average, interviews lasted about 30–45 min.

### Data management and analysis

All interviews were conducted via telephone and online platforms and were all digital voice recorded. The records were later transcribed verbatim. All data was de-identified and stored separately. Deductive content analysis was done manually, by teams across the 4 countries through interactive weekly virtual meetings during the process of data analysis. Initially the Ugandan team comprising of four members identified emerging major themes and codes for the transcripts. Codes corresponding to financial and non-financial incentives and dis-incentives were identified which were shared for guiding other country teams. This allowed for deductive and iterative exploration and assignment of the codes. Categories and themes were generated according to the study objectives. Appropriate quotes were used to support the findings and to capture variation across the countries. Two to four researchers coded interviews across the countries based on the number of interviews conducted.

## Results

### Description of the key informants

Across the four countries, 60 key informants were interviewed 18 of whom were policy makers and 25 health providers (Table 2).

**Table 2:** Summary of the key informants

	DRC	Nigeria	Senegal	Uganda	Total
**Policy makers**	5	2	05	6	18
**Health providers**	4	3	10	8	25
**Health Managers**	3	2	05	7	17
**Total**	12	7	20	21	60

### Health workforce incentives

The analysis categorized health worker incentives into two thematic areas: (i) financial and (ii) non-financial incentives, with the accompanying dis-incentives. The financial incentives included allowances, temporary tax exemptions, and salary increments, while non-financial incentives included services re-organization, supplies augmentation and increased staffing among others. The modalities through which some of these incentives were implemented also led to dis-incentives as described below.

### Theme 1: Financial incentives and dis-incentives for health workers

Financial incentives in the form of allowances and salary increments were provided for health workers to mitigate the effects of increased workload, working hours, and other psychological impacts during the COVID-19 response across the four countries in 2020/2021. The financial incentives were delivered under varying modalities and the way they were implemented generated dis-incentives in some settings (Table 3).

**Table 3:** Summary of financial incentives provided for health workers and the dis-incentives during the COVID-19 response across the countries

Financial	Incentives	Dis-incentives
**Salaries**	Temporary tax exemptions	Inadequate payments
	Salary increments	In consistent payments
**Allowances**	Risk/hazard	Unfair remuneration
	Overtime	Delayed payments
	Activity specific (e.g. contact tracing, testing etc.)	Equal payments with those in positions perceived to be less hazardous.

### Allowances, salary increments and short-term tax exemptions

Health managers and workers in DRC, Nigeria, Senegal, and Uganda reported that health workers received monetary benefits in the form of salaries and allowances as a means of motivation for their effort towards the continuity of health services during the COVID-19 response. In Senegal, it was mostly financial motivation. They reported that from March 20 to December 20, there was a financial incentive of one hundred and fifty thousand FCFA (150,000 FCFA or 240 USD). In the DRC, the salaries of health workers involved in testing for COVID-19 was similar to all other staff involved in the response in any committee (epidemiological surveillance, case management, communication, etc.) but a temporary suspension of taxes was used as an incentive for health workers during the pandemic.

*“… the government decided to temporarily remove the tax-fee deducted from the remuneration of health workers of the public health system for two months to compensate for the lack of payment for overtime due to the increased workload resulting from the COVID-19 pandemic.”*(KII, DRC).

*“To my knowledge like I said earlier, health workers were paid some COVID-19 allowance for three months, which was a proportion of their salaries depending on where they work,, your grade, or level of employment.”* (KII, Nigeria).

*“There is the financial motivation that we gave but also the equipment…., the endowment of materials. It even goes beyond individual motivation”* (KII, Senegal).

*“We heard allowances were paid according to the number of days someone worked…allowances for those ones working in the Corona treatment unit…and then, we also had allowances of course for anybody exposed” (*KII, Uganda).

However, since financial incentives were mostly administered in an unstructured/ad-hoc manner, some health workers felt they were unfairly distributed and complained about the lack of transparency in the allocation of these incentives. In Nigeria, it was reported that payments did not meet the health worker expectations, while in Uganda it was reported that allowances were given selectively to some health workers such as those involved in contact tracing, COVID 19 testing, and COVID 19 isolation units but not to others. In the DRC, there were concerns about delayed payments and the fact that the payment of health workers involved in testing for COVID-19 did not differ from other staff involved in the response in other committees such as epidemiological surveillance, case management, or communication. The key informants revealed that although allowances were availed, there was a sense of dissatisfaction due to delays and some health workers not getting paid as quoted below.

*“The government has been giving allowances to staff who are working in isolation units. Although I do not know about other hospitals because I’ve heard people complain that some of them have not been getting allowances.”* (KII, Uganda).

*“But regarding the incentives, this is where the problem lies, because last week we were paid for the month of May and June of last year. The Government recognizes this and only keeps promising.”* (KII, DRC).

*“There were some issues around special allowances for frontline workers in COVID-19 response. The allowance wasn’t provided as the workers expected but to some extent, it was provided so that’s just it.”* (KII, Nigeria).

### Non-financial incentives and dis-incentives

The non-financial incentives reported across the countries included those that were related to COVID-19 management such as capacity building, safety and risk protection, welfare including recognition and service re-organization (Table 4). Services re-organization included re-organizing of service delivery points, and re-scheduling of services and was done to protect health workers from work overload and reduce contact with potentially infected clients. There was also the recruitment of additional staff on contractual short-term basis. These were perceived to positively affect motivation and performance of health workers. However, these strategies also generated counter effects on workforce motivation including discontent as described below.

**Table 4:** Summary of Non-financial incentives provided to health workers and the disincentives across the countries

Non-financial	Incentives	Disincentives
**Capacity building**	- Training (telemedicine training, face to face amidst SOPs guidelines observance)	Rapidly changing guidelines, Virtual meetings
	- Supervision	
	- Mentorship at group and individual levels	
	- Workshops	
**Safety and risk** **Protection**	- Protective gear medical supplies like PPEs, sanitizers	Inadequacy of protective gear, Restriction of PPEs to only COVID 19 facilities
	- Priority COVID-19 Testing for Health workers	
	- Treatment (Medical care)	
	- Vaccination	
	- Health insurance	
**Welfare**	- Meals	Variably implemented, not for all.
	- Transport	
	- Accommodation	
	- Psychosocial support/ counseling	
	- Health education	
	- Recognition	
**Service delivery re-organization**	- Rescheduling selected services to specific clinics	Work overload
	- Task shifting to Community Health Workers	
	- Recruitment of additional staff	
	- Designation of facilities to care for COVID-19 cases	

### Capacity building

#### Training for health workers

Across the four countries, efforts were made to strengthen and optimize the skills and performance of health workers particularly regarding COVID-19 management and infection control practices such as environmental cleanliness and disinfection and PPE protocols. Trainings in infection control were perceived to make the workplace safer and a motivation to the continuity of health care services provision as mentioned by some respondents:

*“…like I said every health worker has been trained on protection, prevention, control and how to protect themselves. They have been provided with the necessary equipment to protect themselves”* (KII 26, Nigeria).

*“…From the first day, we did an accelerated training on how to wear PPE and gowns, and how to wash our hands and the circuit that we must do. It was first a training problem that we first faced.”* (KII, Senegal).

In DR Congo, several trainings were offered to health workers cutting across hygiene and sanitation to clinical training for health professionals. DRC also used a variety of training approaches including virtual training to cover aspects of hygiene and sanitation but also to ensure continuity of services using approaches conducive to the demands of the pandemic.

*“The following trainings and capacity building workshops were organized on the disposal of waste and used family planning supplies;…. integrating new approaches into community-based services; on COVID-19 transmission prevention in maternity wards; on initiation of breastfeeding and exclusive breastfeeding in the context of COVID-19; and on the COVID 19 nutrition management protocol.”* (KII, DRC).

#### Supervision and mentorship

During the pandemic, the ability to have online supervision and to work from home was an incentive because it ensured that only the most critical staff needed to report on duty to minimize infection risks. In Uganda for instance direct supervision from those in higher levels of management was perceived to be beneficial.

*“…we have ward managers that actually supervise these workers. So, we had direct supervision right from the top management that is the director”* (KII,Uganda).

*“During COVID-19, with online work, we are the department head, we ensure the minimum service. Our subordinates worked online, and they reported to us on their personal work daily.* (KII, DRC).

*“Yes, those people would be trained in IPC and patient management and I would personally mentor them as a unit in-charge.,….I would ensure a balance of seniority for example I pair those who have been there for a month or two with a new person.”* (KII, Uganda).

### Safety and risk protection

#### Provision of COVID-19 management medicines and supplies

Respondents across the countries revealed that efforts to provide sufficient quantities of medical supplies and sundries were crucial for COVID-19 management as one of the most compelling incentives as noted below:

*“…Usually when people mention motivation, they think of financial rewards but what I have seen is that if I have the right equipment, I have the right sundries [and] resources I can do my work without having to plead for gloves or masks, and that alone gives me reason to go and work. Even without thinking of government is going to pay or not pay. I have had staff here manage COVID-19 and we have not paid them a coin.”* (KII, Uganda).

*“There is the financial motivation that we gave but also the equipment, the endowment of materials. It even goes beyond individual motivation.”* (KII, Senegal).

*“The equipment are full, I think you have just seen a container that has just arrived, I think the container has been deposited there, this container is to support the prevention of infection.”* (KII, DRC).

#### Prioritizing health worker medical care

Deliberate efforts were made to monitor the well-being of healthcare workers who were involved in the COVID-19 response. In Uganda, Nigeria and Senegal, this included continuous screening of health personnel to identify those infected, those who fell sick and those who died in order to prioritize them for vaccination and treatment so as to reduce their vulnerabilities. Prioritizing COVID-19 testing, and treatment were some of the incentives provided to motivate health workers in DRC and Uganda as part of the countries’ response to COVID-19. Health workers were tested for COVID-19 regularly, and priority treatment and isolation were given to those who tested positive for COVID-19. Senegal had policies and guidelines that provided a framework for providing priority health services to health care workers who become ill because of engagement in a public health emergency response and shown in some of the quotes:

*“Health workers were prioritized for free testing…Yes, we have been tested free of charge of course… When the vaccines came, the very first group were the health workers. This was a national pronouncement and a guideline which health workers had to abide with and comply.”* (KII, Uganda).

*“…They were also tested regularly in case of suspicion and were taken care of in case of confirmation. In case of death, funeral expenses were also covered”* (KII, DRC).

#### COVID-19 vaccination for health workers

Across the four nations and at the beginning of vaccination campaigns, the limited quantities of vaccines meant that the vaccination of health workers was prioritized because of their higher risk of exposure at the frontline of the response.

*“The country first sensitized health care workers to get vaccinated. This is why the majority of health workers are vaccinated. In turn, we have also strengthened the capacities of the community health workers; we have sensitized them to be vaccinated”* (KII, DRC).

*“….But also when it comes to vaccines, we started by vaccinating health personnel”* (KII, Senegal).

In Senegal, the government prioritized vaccinating health personnel and the elderly and people with comorbidities, i.e. 3% of the population [52].

### Welfare-related non-financial incentives

#### Welfare support for health workers

Efforts made to improve the welfare of health workers during the COVID-19 response included the provision of meals, housing, and transport in countries like Senegal and Uganda.

*“…the transport of medical students who came in addition to the doctors out of service, now we have made efforts; plus housed and transported them”* (KII, Senegal).

*“…They were feeding all health workers and they would bring lunch; posho (corn meal), rice and beans which they would serve to the doctors at the hospital, they did that for close to three months. And another thing is these guys gave transport to our staff because remember during the COVID-19 response, there was a total lockdown….and you know government hospitals cannot house 50% of the staff…actually I think they offered two buses to be transporting staff from their homes to the hospitals every single morning and evening* (KII, Uganda).

*“…We had to identify a house to provide extra accommodation for our staff who were working so much and we did not want them to travel to their homes to transmit the disease to their family members.”* (KII, Uganda).

#### Provision of psychosocial support

Across the four countries, frontline healthcare workers caring for patients in intensive care units (ICUs) faced extreme pressure, from extra workload and this was exacerbated whenever their own colleagues succumbed to COVID-19 infection. Stress was reported from multiple sources including the loss of patients and other challenges for which they received psychosocial support as a coping strategy. This was delivered through counseling, meetings, and health education.

*“… Like in my facility, there is no way you could be absent because the work was too much and the staffing was low so your absence would be noticed.”* (KII, Uganda).

*“Yes, there is psychosocial support for health workers at the Nigeria Center for Disease Control (NCDC)… I think they had a place where you can talk to somebody if you are feeling the weight of the response.”* (KII, Nigeria).

**“***The first wave of COVID-19 was very stressful. I frequently tested for COVID-19. Before the results were announced, I was receiving psychosocial care.”* (KII, DRC).

*“The facility managers or in-charges also had sessions where presentations were made to them…we also had social workers among the psychosocial staff….time to time in our hospital every Wednesday we have a meeting for the senior….in such meetings we have the psychosocial officer….and then we are counseled. Then we also have staff who tend to panic a lot we also call the psychosocial officer to talk to them…there were both individual (one on one) and group counseling sessions done…whenever need arose.”* (KII, Uganda).

*“…some of (the health workers) got scared; we had many staffs who were sick, about fourteen were sick of COVID-19 discouraging most of the others but we had to morale boost them, support them by counselling by continuity of communication.”* (KII, Uganda).

In DRC, a standard operating procedure was developed and provided for the cascade training of psychosocial agents, the organization of psycho-educational sessions for patients and contacts, and the organization of psychosocial support for personnel involved in the response. In Senegal, at the start of the pandemic, simulation exercises were used to get teams prepared psychologically. Discussion groups were organized in the treatment centers and these structures benefited from the support of the psychosocial unit of the Ministry of Health, which provided support supervision for the health workers. During the second wave, this psychological support was not provided.

*“…we must already continue to make staff aware of the presence of the pandemic, which is still there. Second thing, you have to do simulation exercises. At our level, we do a guard maneuver every day, a person who takes guard. Every morning after guard duty, this team does a guard maneuver and sometimes we take the COVID-19 theme with the example of a person with COVID-19 or a person who died of COVID-19 and see how the body should be handled. We do exercises and it is to push the staff to become better acquainted with these different forms of intervention because we are not used to doing it. So it’s the sensitization so that the guys are psychologically ready, then it’s the simulation exercises and after the simulation exercises we come to the material preparation with the verification.”* (KII, Senegal).

#### Provision of health insurance

Differences in the provision of health insurance to health workers were noted across the countries. Life insurance was provided in Nigeria as part of the compensation package during the COVID-19 response. However, in Uganda with no insurance measures in place to protect public health workers in pandemic response situations, only contracted health workers had access to health insurance.

*“The health workers that were insured were those not on the government payroll.…The government payroll system is a bit different, for those who were not there, they were insured, then of course those who were enrolled during the response were not insured and that is the truth”* (KII,Uganda).

In Senegal, according to the workforce management plan, additional incentive measures such as health insurance covering the treatment of COVID-19, sickness allowances for workers who either contracted the virus or self-isolated due to close contact with infected workers, and payment in the event of death were put in place [53].

In the DRC, health care workers generally did not have full coverage of health insurance. The government provided a bonus to health workers that unfortunately did not cover all their social needs.

*“… No, the government has only paid the premium for management of the sick. The COVID-19 management of the inputs had to be bought by the health worker.”* (KII, DRC).

*“I believe that the problem of financing in the health sector remains. Generally, the government supported our structures in terms of bonuses granted to us and certain benefits that we derive as agents of the state, but the effective implementation of activities requires support from partners.”* (KII, DRC).

### Recognition

Our analysis revealed that some health workers received recognition for the effort they invested into the COVID-19 response in countries like Uganda and DRC. Appraisals were common and recognition of outstanding performers during functions or in the event of officials coming around the health facilities:

*“…we recognize best performers…we tried to recognize them, especially whenever we had officials coming in, we kept appraising those who have really performed well in front of them the officials” (*KII, Uganda).

In the DRC, the president of the republic recognized the work done by health workers during the 35th Council of ministers and decided to reward their efforts and bravery with an additional bonus on the occasion of the 60th anniversary of the country’s independence.

“*These kinds of bonuses must continue. Not just for a month, but continuously*…” (KII, DRC).

However, there was a sense of dissatisfaction because these recognitions were perceived to be unfair.

*“Yeah people were recognized although not uniformly”* (KI, Uganda).

### Service delivery re-organization

Across the four countries, multiple strategies were taken to de-congest facilities and reduce infection spread. These included the re-scheduling of selected services to specific clinic days, task shifting to community health workers, recruitment of additional staff and the designation of facilities to cater for COVID-19 cases (Table 4).

In Senegal, particularly in Thies, there was a re-organization of services:

*“…in fact, it was the internal medicine department that was amputated, we had two units in the internal medicine department; the unit of men and that of women; so, when COVID-19 emerged,…. it was the women’s unit which was more accessible and more isolated, and we transformed it into a treatment center.* (KII, Senegal).

*“…before (during the early COVID 19 period), the COVID-19 centers were established in the mental health clinics, hospital administrators were asked to work with the lower level health units to establish alternative mental health isolation units”* (KII, Uganda).

In the DRC, some health programs were affected by COVID-19 because of the minimum service requirements established to deal with the pandemic. Some organizations utilized additional human resources, but others were not able to obtain additional workers.

*“…There has been an impact because in the health zones, the same community relays who sensitize parents to come and vaccinate their children in the health care facilities have been reassigned to go and do sensitization against on COVID-19, which has resulted in a decrease in facility personnel for the vaccination activity. ”…* (KII,, DRC).

However, because of shifting services to other facilities, health workers had to be re-deployed from COVID-19-designated facilities to other peripheral facilities resulting in longer travel routes which was a disincentive to them.

### Recruiting additional staff

All countries made efforts to co-opt additional staff during the pandemic. Uganda and Senegal recruited health professionals while DRC recruited volunteers. The Ugandan government in partnership with development partners, made available a budget to recruit additional health workers to serve especially in hard-pressed settings. Advertisements sent out on April 27th 2020 and July 27th 2021 for health workers resulted in 31 and 70 contracted workers respectively with payments for a period of 6 months in the different years mentioned [54, 55]. Overall, around 700 health workers were hired in Uganda during the COVID-19 emergency response. These included anesthesiologists, laboratory professionals and nurses among others. In Senegal, the government initially requested for health personnel, and recruitment of health personnel on contractual basis for varying periods of time and these were paid by the government to bolster the numbers available to provide services:

*“To overcome this difficulty of lack of staff, the hospital at the beginning had requisitioned doctors, nurses to come and help us. But afterwards, we were forced to recruit outright especially the Ministry which had to recruit staff at the level of the Emergency Treatment Centre and make fixed-term contracts of three months, six months, and 1 year for some so that we could have more staff.”* (KII, Senegal).

In DRC, the recruitment of additional health workers for the COVID-19 response was focused on volunteers until a specific number was reached per commission and level. But the leadership of the commissions was under officers experienced in the response.

“*I presented myself as a volunteer at the start of the COVID-19 pandemic (March 2022), I was recruited very easily…”* (KII, DRC).

*“Recruiting is too hard, but we resorted to the example of medical schools and universities all over our provinces that train doctors, so since everything was already paralyzed, we had to resort to these people so that they could quickly help to minimize the situation.”* (KII, DRC).

Unfortunately for Uganda, the contracts for staff recruited during the COVID 19 pandemic were terminated approximately one year later in 2022 due to depletion of personnel budgets [56]. This created a sudden gap in numbers and skills of health workforce as well as despondency among contracted staff as noted by a key informant in quote;

*“… Right now, the staff don’t even know if they’re going to be absorbed within a government system or if they should start looking for other jobs because the contract is expiring in July. There was a validation exercise where they came to check whether these staff are actually working. They interviewed one by one, but that was in December and till now in February we have not got any kind of communication.”* (KII,Uganda).

## Discussion

Health systems in Africa have long been plagued by weak institutional and human resource capacities, health worker migration, inadequate incentives, and un-coordinated development support, among others. Experiences gleaned from the COVID 19 amidst chronically weakened health systems emphasize the need to anticipate, enhance incentives, and exercise due precautions in order to reduce dis-incentives during emergencies.

In our study health worker incentives during the COVID-19 response were mostly unplanned, predominantly non-financial, and invariably implemented. Across these countries there were neither guiding frameworks nor standard pre-determined packages of financial and non-financial incentives for health workers during emergencies. Consequently, each country implemented varying incentive strategies during the COVID-19 pandemic. The financial incentives provided for health workers in the form of allowances and enhanced salaries to mitigate the effects of increased workload and risks were appreciated. However, these tended to generate discontent due to their perceived inadequacy, unfairness in their implementation, and delayed remittance among others. The real or perceived inconsistencies and the lack of transparency in the way incentives were provided resulted in a degree of dis-incentivization for the health workers who opined that things should be planned better. Moreover, the non-pharmaceutical interventions implemented to reduce the spread of COVID-19 such as lockdown, quarantine and isolation, further exacerbated the health worker stress by compromising their mobility. This study reveals that within these already weakened health systems, poorly implemented incentives may easily erode motivation of health workers more so during emergencies.

Health workers allude to the necessity to access the basic environmental amenities as a baseline to committed and satisfactory work. During emergencies, the absence of basic supplies in the workplace creates a scenario where health workers experience heightened stress and feel oppressed because their ability to work safely and productively is already constrained.

The broad scope of non-financial incentives included the provision of medical supplies for management of COVID-19 in the form of PPEs, sanitizers, equipment, training of health workers, medical care, free testing, vaccination, welfare in the form of free meals, transport, housing, psychosocial support, recognition, and supervision although these were not equitably spread across the four countries. The provision of medical supplies for management of COVID-19 in the form of PPEs, sanitizers, equipment and others was perceived as a motivator for the continuity of health services across the four countries. This gave health workers a sense of protection and confidence to manage COVID-19 cases and other patients with minimal fear of infection. Similar findings have been reported in Zimbabwe, Kenya, Egypt, Uganda and South Africa [57, 58]. It is pertinent to note, however, that the primary goal of providing PPEs is not to motivate health workers but rather to prevent cross-infection [59]. In any infectious disease epidemic, PPEs should be availed as a matter of urgency. However, this was not always the case especially in resource limited settings where rationing is prevalent and stock outs are frequent, and PPEs may be deemed to be a privilege rather than an entitlement [60]. Moreover, in some settings PPEs were predominantly provided to those facilities managing COVID-19 cases yet health workers all over the country perceived themselves to be at a heightened risk of infection because they engage with all sorts of patients before they are diagnosed. This too was a major dis-incentive to the health workers in the non-COVID treatment facilities because they felt that governments were placing them at undue risk. In this kind of environment, even the provision of financial incentives may not yield the anticipated benefits. Other non-financial incentives implemented such as, welfare (transportation), recognition and systems re-organization such as the re-organizing of service delivery points and re-scheduling of services among others decongested health facilities and supported health workers. They, however, also created dissatisfaction because they were perceived to be haphazardly implemented. Moreover, these incentives were variably implemented across the countries highlighting the need for further study around their potential value and detriment to the system during emergencies and perhaps to inform future planning.

Governments bracing for future infectious outbreaks should consider enhancing capabilities for the local production of PPEs and other related supplies which are critically needed as demonstrated countries during the COVID-19 pandemic [61]. Across countries, governments partnered with other stakeholders including the WHO and implementing partners, like Baylor, Infectious Diseases Institute, UNICEF, and other private organizations to provide incentives. However, these partnerships and roles were largely uncoordinated. A similar situation was reported during the Ebola outbreak where the WHO, Africa Centers for Disease Control and Prevention, UNICEF, the Red Cross movement and other international and local partners, donors, researchers and communities worked hard to end the Ebola outbreak in West Africa [62]. The critical need for strategic partnerships to enhance health workforce before and during emergencies cannot be overemphasized. So is the need for strong and coordinated alliances between governments, pharmaceutical companies, and manufacturing companies to anticipate and meet demands to mitigate some of the restrictions relating to supply chain, costs, and procurement procedures.

The COVID-19 pandemic was characterized by unpredictable surges in cases which enormously constrained health systems globally [28], particularly in Africa with already weakened health care systems and a shortage of critical care specialists and equipment [63]. Health workers as first responders were more at risk of contracting the disease [64]. In fact a number of health workers died in the event of saving others [11]. The loss of colleagues within the workforce has previously resulted in fear and stress [65] and in worst cases abscondment from duty [66]. It is encouraging that across the study countries, governments and other stakeholders prioritized regular and cost-free testing for health workers to prevent infection and mitigate the potential loss of health workers which could have had deleterious effects on other workmates. Across the four countries, life insurance was only provided in Nigeria and this motivated health workers during the COVID-19 response. The lack of medical and even life insurance for the health workforce is a huge oversight because health workers experience increased morbidity and mortality during disease outbreaks. This is because more often than not, these uninsured health workers also happen to be at the frontline of the response [67]. Available evidence has shown that health workers have lost their lives during epidemics without being compensated by their governments [68]. Brocardo (2017) affirmed health insurance as an enhancer to health worker performance in Ethiopia [69]. Most countries like Uganda and DRC have no policy on providing health insurance for health workers during emergencies. Consequently, health workers that were insured during the response were those who worked with other organizations, and private practitioners but not public sector workers and those that were non-gratuitable. There is a need to develop policies that cover both health and life insurance for health workers in low-income countries especially during epidemics. For Africa, which has had a 63% increase in diseases spread from animals to humans seen in last decade [70], these policies are long overdue [71]. In addition to instituting policies on health and life insurance, countries should consider providing more benefits to health workers such as covering out of pocket costs during emergencies in addition to prioritizing health workers for treatment and psychosocial support.

In Uganda, Senegal and Nigeria, welfare support was offered as an incentive in the form of free food, transport, and housing. Similar findings have been reported in the United states where food, transport, housing were offered as incentives during the COVID-19 pandemic response to health workers [72]. Health worker incentives like housing in Lesotho, Mozambique, Malawi and Tanzania; staff transport in Lesotho, Malawi and Zambia and free food in Mozambique and Mauritius had been reported to address social needs of health workers [73]. In Tanzania for instance, the provision of housing allowance to health personnel was noted to improve the quality of care through compliance to Integrated Management of Childhood Illness (IMCI) guidelines in Tanzania [74].

By virtue of the heightened anxiety during disease outbreaks, the need to provide psycho-social support for health workers cannot be overstated. Across the countries, psychosocial support was offered to frontline health workers especially the COVID-19 Treatment Unit (CTU) staff. This was in the form of counseling, meetings, and health education. Through these avenues, health workers would share concerns and obtain emotional support that enabled them to continue in the line of duty amidst the prevailing challenges. The value of social and psychological support through interpersonal interactions during the COVID-19 response in Africa has been echoed by [75, 76]. Additionally, psychosocial support allowed for continuity of service by health workers during the Ebola epidemic in Sierra Leon [77]. More emphasis is needed to practically boost the psychosocial support component highlighted in the COVID-19 response guidelines for better pandemic response in the future. In settings where health workers are already overworked, underpaid and de-motivated, improvements in their welfare and psychosocial during outbreaks and other emergencies should be a matter of priority.

The recognition of health worker performance was reported to motivate key front line health workers in Uganda although the pandemic restrictions hampered adequate recognition in some public hospitals. In other countries, public recognition was not reported as a key motivator during the pandemic response due to COVID 19 public gathering restrictions. Relatedly, other studies have reported recognition to influence of health workers motivation [69]. It is therefore important to institute regular schedules to recognize outstanding health workers and more so for notable efforts during epidemic because this enhances health worker motivation.

Training in IPC and COVID-19 management, although a necessary component of epidemic management, was seen to inspire health workers who were involved in the response across the countries. The provision of training has also been reported to be a great spur to health worker efforts during the COVID-19 pandemic in Serbia [26]. Furthermore, the COVID-19 pandemic highlighted the value and necessity of evolving alternative approaches to training including virtual/online training or tele-applications to build the capacity of health personnel and to supervise them. The utility of these beyond the COVID-19 pandemic need to be evaluated and sustained [78]. However even more critical is the need to anticipate, provide training, and certify health workers prior to outbreaks. Certification of training has also been revealed to be a great motivator for health workers [79].

In Uganda, response team leads and hospital managers monitored the performance of staff in CTUs on IPC measures and their presence at work. Health worker supervision also aided the management of bonus payments because health workers in CTUs in Uganda were paid according to the extra days worked. A systematic review in low- and middle-income countries highlights the importance of supervision to boost the performance of health workers [80]. Supervision during epidemics enhances health worker motivation because it makes work enjoyable, pleasant, and calm [81].

The implementation of financial incentives tended to vary across the four countries. Incentives provided ranged from nothing at all beyond the usual stipulated salary, to intermittent allowances, to carefully calculated allowances based on extra days of work. Several countries reported that some cadre got allowances for instance workers at testing stations and ICUs in Uganda, while others at treatment facilities received no additional payments. It appeared that none of the countries had in place any guidelines on how to provide incentives for health workers during epidemics. The reports of perceived unfair provision of allowances were rife across all countries exposing a gap in the countries to foster the predetermined fair and accountable provision of incentives. The challenges posed by the lack of guidelines on incentives during emergencies have not only been noted in Africa. In Europe there were calls to harmonize guidelines around incentives for health workers when it was realized that health workers were often under immense pressure to care for severely ill patients with a new disease, under strict hygiene conditions and with lockdown measures creating practical barriers to working [27].

The value of providing financial incentives during disease outbreaks cannot be overemphasized because during epidemics, health workers encounter work pressures beyond routine service delivery. In the European Union, most countries instituted financial incentives. These were intended to support health workers and enable them to do their job. These included additional financial support and compensation above normal salaries to health care workers involved in the COVID-19 response, a lumpsum payment to families in the event of death of a health worker following COVID-19 infection, allowances to cover childcare costs during the crisis in the case where a health worker’s partner could not take paid leave, and temporary salary increment [27]. It is reported that in Sierra Leon during the Ebola epidemic, risk allowance motivated health workers [25]. Most financing for emergencies focusses on administrative and service delivery enhancement with little or no attention given to incentives to boost health worker motivation. Digital payments have been proposed as an avenue to streamline payments of health workers and could be an option to consider during emergencies.

Although a diverse package of incentives was implemented across the four countries, inconsistencies, lack of transparency, perceived inadequacy and inequity of incentives were reported in the study countries as has been elsewhere in Africa [22]. In Nigeria and Uganda, the phenomenon of some health personnel missing out on financial incentives following irregularities in payment of allowances has been reported in other studies [22, 33]. There is therefore a need to develop guiding frameworks within which governments and partners can deliver incentives and reduce dis-incentives for the health workforce during emergencies.

The study was qualitative implying the extent of the health worker incentive strategies and experiences around the incentives cannot be quantified. Future studies could reflect on measuring these aspects. We obtained information from various stakeholders including health managers, policy makers and health workers which allowed for triangulation of the findings.

## Conclusion

Health related emergencies call for incentive packages to stretch beyond those normally provided because of the additional risks and stress encountered by the workforce operating amidst already weakened health systems. The elevated risks and heightened workload necessitate the mandatory provision of safety gear and adequate supplies. Although important, financial and non-financial incentives may end up being dis-incentives if perceived to be unfair in their implementation. Financial and non-financial incentives should preferably be pre-determined, equitably and transparently provided during health emergencies because arbitrarily applied financial and non-financial incentives become dis-incentives. Moreover, financial incentives are useful only as far as they are administered together with non-financial incentives such as supportive and well-resourced work environments. The potential negative impacts of interventions such as service delivery re-organization and lockdown on health worker motivation need to be further interrogated and due precautions exercised to reduce unintended consequences on the workforce during emergencies. Governments need to develop guidelines on incentives during emergencies with careful consideration of mitigating potential dis-incentives. The harmonization of roles across state and non-state sector players in incentivizing the health personnel during health emergencies is paramount.

### Electronic supplementary material

Below is the link to the electronic supplementary material.

Supplementary Material 1

## Data Availability

The notes from the key informants are available on request from the corresponding Author.

## Abbreviations

COVID-19: Corona Virus Disease of 2019
CTU: COVID-19 Treatment Unit
DRC: Democratic Republic of Congo
UNICEF: United Nations Children’s Emergency Fund
CHAI: Community HIV/AIDs Initiative
ICU: Intensive Care Unit
KII: Key Informant Interview
PPE: Personal Protective Equipment
UCAD: The Cheikh-Anta-Diop University
WHO: World Health Organization
