# Supplementary material for: Health workforce incentives and dis-incentives during the COVID-19 pandemic: experiences from Democratic Republic of Congo, Nigeria, Senegal, and Uganda
Source: BMC Health Serv Res. 2024 Apr 3;24:422. doi: 10.1186/s12913-024-10822-6 (PMC10993439; doi:10.1186/s12913-024-10822-6)
Supplement: Supplementary file 1 — Supplementary Material 1 [file 12913_2024_10822_MOESM1_ESM.docx]

**Health workforce incentives and dis-incentives during the COVID-19 pandemic: Experiences from Democratic Republic of Congo, Nigeria, Senegal, and Uganda**

**Key Informant Interview Guide for Health systems Readiness – Human Resources for Health**

1. To what extent did the country experience human resources for health challenges during the pandemic? What was the nature of these challenges?
2. How did the country address HW shortages and capacities during the pandemic? Probe for re-deployment, CHWs, task shifting? Re-training?
3. What role did CHWs play in expanding health workforce capacity during the pandemic? How can they be prepared for this?
4. What mechanisms did the country use to track the health workforce in terms of availability? Vulnerability to infection? Training needs, morbidity? Mortality?
5. How did the country attempt to protect the health workforce against COVID infection, work overload, psychosocial effects of the pandemic? NPIs?
6. Who were the key players/partners in strengthening/supporting the health workforce for the COVID response and what did they do?
7. What efforts did the government put in place to incentivize/motivate the health workforce? Probe for allowances, priority testing, vaccination, insurance? Transport? Recognition? Meals? To what extent did HW get access to these incentives?
8. From your perspective what 3 things in order of priority does your country need to do to ensure that its health workforce is ready for the next pandemic?
